# A Novel UPLC-MS/MS Method Identifies Organ-Specific Dipeptide Profiles

**DOI:** 10.3390/ijms22189979

**Published:** 2021-09-15

**Authors:** Elena Heidenreich, Tilman Pfeffer, Tamara Kracke, Nils Mechtel, Peter Nawroth, Georg F Hoffmann, Claus Peter Schmitt, Rüdiger Hell, Gernot Poschet, Verena Peters

**Affiliations:** 1 Centre for Organismal Studies (COS), Metabolomics Core Technology Platform, Heidelberg University, 69120 Heidelberg, Germany; elena.heidenreich@cos.uni-heidelberg.de (E.H.); mechtel@stud.uni-heidelberg.de (N.M.); ruediger.hell@cos.uni-heidelberg.de (R.H.); 2 Centre for Paediatric and Adolescent Medicine, University Hospital Heidelberg, 69120 Heidelberg, Germany; tilman.pfeffer@med.uni-heidelberg.de (T.P.); Tamara.Kracke@med.uni-heidelberg.de (T.K.); Georg.Hoffmann@med.uni-heidelberg.de (G.F.H.); ClausPeter.Schmitt@med.uni-heidelberg.de (C.P.S.); 3 Department of Internal Medicine I and Clinical Chemistry, University Hospital of Heidelberg, 69120 Heidelberg, Germany; Peter.Nawroth@med.uni-heidelberg.de; 4 German Center for Diabetes Research (DZD), 85764 Neuherberg, Germany; 5 Institute for Diabetes and Cancer (IDC) Helmholtz Center Munich, 85764 Neuherberg, Germany; 6 Joint Heidelberg-Institute for Diabetes and Cancer (IDC) Translational Diabetes Program, 85764 Neuherberg, Germany

**Keywords:** dipeptides, tissue, biofluids, metabolism, mass spectrometry, UPLC

## Abstract

Background: Amino acids have a central role in cell metabolism, and intracellular changes contribute to the pathogenesis of various diseases, while the role and specific organ distribution of dipeptides is largely unknown. Method: We established a sensitive, rapid and reliable UPLC-MS/MS method for quantification of 36 dipeptides. Dipeptide patterns were analyzed in brown and white adipose tissues, brain, eye, heart, kidney, liver, lung, muscle, sciatic nerve, pancreas, spleen and thymus, serum and urine of C57BL/6N wildtype mice and related to the corresponding amino acid profiles. Results: A total of 30 out of the 36 investigated dipeptides were detected with organ-specific distribution patterns. Carnosine and anserine were most abundant in all organs, with the highest concentrations in muscles. In liver, Asp-Gln and Ala-Gln concentrations were high, in the spleen and thymus, Glu-Ser and Gly-Asp. In serum, dipeptide concentrations were several magnitudes lower than in organ tissues. In all organs, dipeptides with C-terminal proline (Gly-Pro and Leu-Pro) were present at higher concentrations than dipeptides with N-terminal proline (Pro-Gly and Pro-Leu). Organ-specific amino acid profiles were related to the dipeptide profile with several amino acid concentrations being related to the isomeric form of the dipeptides. Aspartate, histidine, proline and serine tissue concentrations correlated with dipeptide concentrations, when the amino acids were present at the C- but not at the N-terminus. Conclusion: Our multi-dipeptide quantification approach demonstrates organ-specific dipeptide distribution. This method allows us to understand more about the dipeptide metabolism in disease or in healthy state.

## 1. Introduction

Blood and tissue dipeptides may originate from dietary intake and endogenous metabolism, i.e., protein degradation and synthesis [1]. Intestinal dipeptide uptake is mediated by peptide transporters (such as SLC15A1/PepT1 and SLCA2/PepT2 [2,3]). Dipeptide appearance in blood depends on their affinity to the peptide transporters [3,4] and on their resistance to hydrolysis [5,6]. Hydrolysis of dipeptides into amino acids is mediated by various peptidases [5,6]. Synthesis of dipeptides has been demonstrated for the two histidine dipeptides anserine and carnosine [7]. Based on the number of available amino acids, the dipeptide pool is potentially large, but surprisingly little information is available. In contrast, the metabolic function of amino acids has been described in great detail and amino acid related diseases are well characterized and respective therapeutic approaches have been established [8,9]. Amino acids are ubiquitous and play a central role in fundamental cell metabolism, contribute to energy production, nitrogen balance, neurotransmitter metabolism, vitamins and hormones [8]. Free amino acids are transported by amino acid transporters (AATs), leading to an organ-specific distribution pattern [8].

To obtain deeper insights into blood and organ-specific dipeptide abundance we established a method of dipeptide quantification using ultra-performance liquid chromatography (UPLC) based separation coupled with tandem mass spectrometry (MS/MS). To increase sensitivity and enhanced chromatographic separation, derivatization of dipeptides with 6-aminoquinolyl-N-hydroxysuccinimudyl carbamate (AQC), also known under the brand name AccQ-Tag™ (Waters), was applied. AQC labels primary as well as secondary amines and is widely used for amino acid analysis [10,11,12]. Via this method 36 dipeptides could be identified and quantified. This approach was applied to 13 different organs, blood and urine of wild type mice on standard diets to reflect physiological conditions and relate these to respective amino acid profiles consisting of 17 amino acids. The dipeptide panel comprised dipeptides with amino acids differing in charge (positive/negative/neutral), in side-chain polarity (acidic/basic polar/non-polar), and in side-chain class (aromatic/aliphatic/cyclic/basic/acid/amide) in either N- or C-terminal position to best represent the putative spectrum of dipeptides.

## 2. Results

Our optimized rapid and sensitive method allowed the simultaneous determination of 36 dipeptides composed of amino acids differing in charge, side-chain polarity and in side-chain class in either N- or C-terminal position.

### 2.1. Method Establishment and Validation

**Figure 1:**
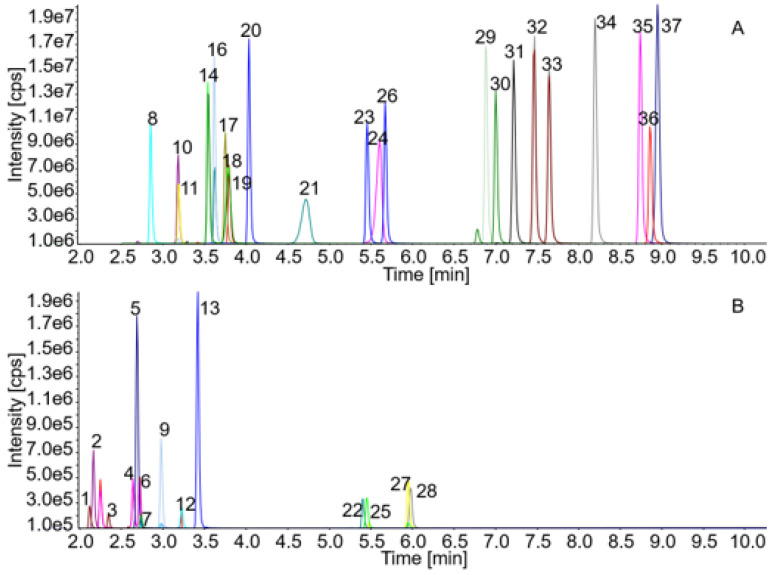
Chromatograms of 36 dipeptides and the internal standard norleucine.0.5 pmol per analyte were loaded on column. (**A**) Dipeptides with higher signal intensity; (**B**) those with lower intensity yields. Compounds are corresponding to the numbers in the chromatograms as follows: 1. Ser-His; 2. Gly-His; 3. His-Ser; 4. Carnosine; 5. Ser-Gln; 6. Ala-His; 7. Anserine; 8. Gly-Asp; 9. Asp-Gln; 10. Glu-Ser; 11. Gly-Glu; 12. His-Ala; 13. Ala-Gln; 14. Ala-Gly; 15. Ser-Ala; 16. Glu-Glu; 17. Pro-Gly; 18. Gly-Sar; 19. Ala-Glu; 20. Ala-Ala; 21. Gly-Pro; 22. γ-Glu-ε-Lys; 23. Ala-Tyr; 24. Ala-Pro; 25. Leu-His; 26. Tyr-Ala; 27. His-Leu; 28. Arg-Phe; 29. Gly-Phe; 30. Val-Tyr; 31. Pro-Leu; 32. Ala-Phe; 33. Phe-Ala; 34. Norleucine (internal standard); 35. Aspartame; 36. Tyr-Phe; 37. Leu-Pro.

Each dipeptide is represented by a specific MRM with the fragment mass 171.1, which is derived from the derivatization reagent AccQ-Tag™. Wherever possible, a second qualifying fragment (Q3) was included. Due to the low mass of some compounds this was not feasible for every substance (Table 1). For clarity, only one specific MRM per analyte is shown in the chromatogram (Figure 1). MS parameters (Q1, Q3, DP, CE, CXP) were optimized for all derivatized dipeptides via manual tuning and flow-injection analysis. Quantification parameters can be found in Supplementary Table S1.

Baseline separation was achieved using optimized chromatographical conditions for the isomeric dipeptides Ser-His/His-Ser, carnosine/Ala-His/His-Ala, Ala-Gly/Gly-Sar, Gly-Pro/Pro-Gly, Pro-Leu/Leu-Pro, Phe-Ala/Ala-Phe, Ser-His/His-Ser, Ala-Tyr/Tyr-Ala and Leu-His/His-Leu (Figure 1 and Table 1). Ala-Leu and Leu-Ala shared the same retention time and could therefore not be discriminated under the applied chromatographic conditions. Cys-Gly showed a high background peak which made correct quantification not feasible. Gly-Leu and Leu-Gly separated well but the chromatograms showed also a strong background peak between the peaks of the two closely eluting substances which made quantification in lower concentrations difficult. Signal intensity (Figure 1) and calibration range (Supplementary Table S2) differ between the types of dipeptides. The LOQ varies between 0.1 and 2.5 fmol depending on the analyte. Dipeptides with multiple amine groups, such as His-containing dipeptides, generally yielded lower signal intensities compared to the majority of dipeptides containing a single amine group.

Intra-assay precision was determined for most analytes with CV values below 10%, His-Ala, carnosine, His-Ser and Arg-Phe were below 20%CV while γ-Glu-ε-Lys showed the highest deviation of 25%CV. Inter-assay precision measurements were performed on three different days, again most analytes delivered a deviation below a CV of 10%. Tyr-Ala, anserine, Tyr-Phe, carnosine, Leu-His, Ala-His, His-Ser were below 20% and Ser-His, Gly-His, γ-Glu-ε-Lys as well as Arg-Phe showed deviation of less than 30% (Supplementary Table S2).

### 2.2. Recovery

Determination of recovery rates was performed by spiking of lung tissue samples with standard substance mixtures of different concentrations. Lung tissue was chosen for the spiking experiments because of the rather low concentrations of dipeptides in these samples. For the highest concentration, recovery rates between 79 and 101% were achieved. All results are listed in Supplementary Table S3. Determination of recovery rates of standard substances was performed by quantification of 1:30 dilutions of three high concentrated standard mixtures in 0.1 M HCl. Recovery rates varied between 75 and 120% and are listed in Supplementary Table S4.

### 2.3. Tissue-Specific Dipeptide Profiles

**Figure 2:**
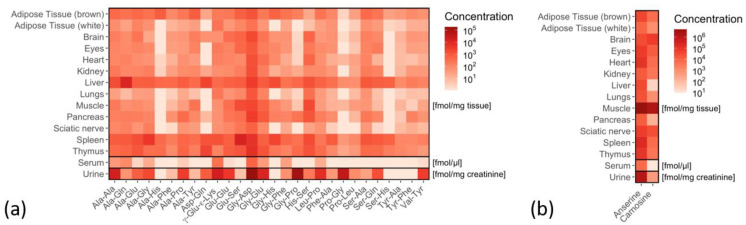
Distribution of dipeptides in murine tissues and biofluids. (**a**) Heat map showing the concentrations for 28 dipeptides and (**b**) carnosine and anserine in murine tissues (*n* = 6, except for urine *n* = 5). Mean values are given in fmol/mg tissue. In serum, the concentrations are given in fmol/µL and in urine in fmol/mg creatinine.

**Figure 3:**
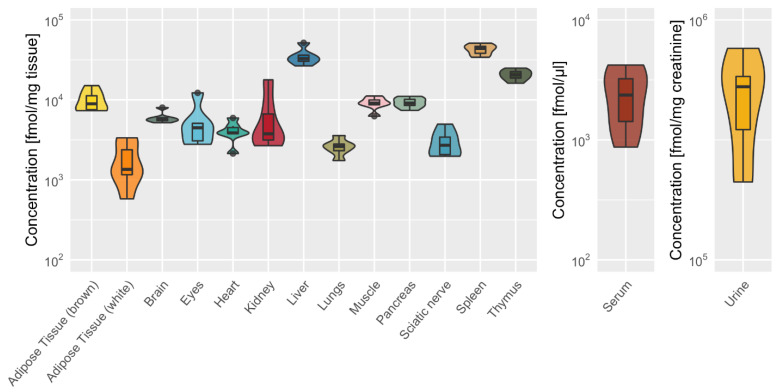
The sum of 28 dipeptides from various tissues and biofluids (as depicted in Figure 2). Sums are presented as violin blot showing median (bold line) with standard deviation (*n* = 6; except for urine *n* = 5). Concentrations are given in fmol/mg tissue in tissue and in fmol/µL in tissue and in fmol/mg creatinine in urine.

A total of 30 of the analyzed 36 dipeptides were detected and quantified in mouse organs (Figure 2 and individual dipeptide concentrations are listed in Supplementary Table S5), and only 6 dipeptides were consistently below the detection limit (Arg-Phe Gly-Sar, His-Ala, His-Leu, Leu-His and the non-natural occurring dipeptide Aspartame). Some dipeptides, such as carnosine, anserine, Ala-Ala, Ala-Gln, Asp-Gln, Glu-Ser, Gly-Asp and His-Ser were present in all organs analyzed, while others (e.g., Pro-Gly, Ser-His) were only detectable in some organs (Figure 2). Anserine and carnosine were the most prominent dipeptides in all organs, with particularly high concentrations in skeletal muscle tissue (Supplementary Table S5). Anserine concentrations were higher than carnosine concentrations in all organs except the brain. Dipeptides were abundant in all organs, serum and urine (Figure 3).

**Figure 4:**
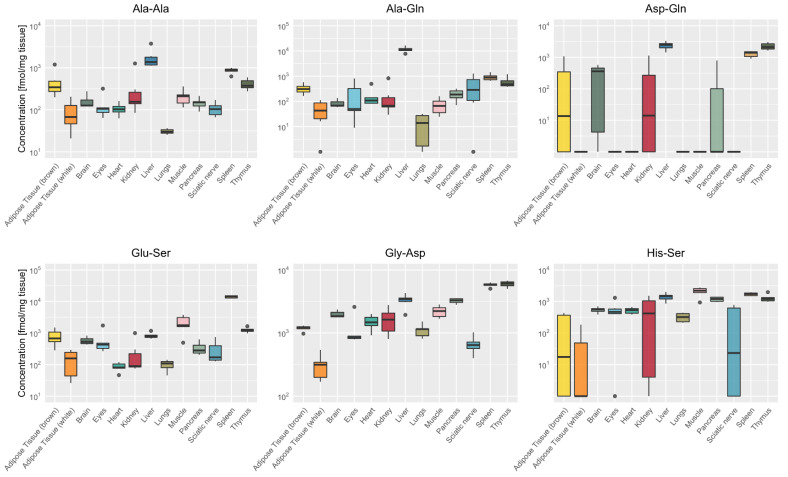
Organ-specific distribution of the six most abundant dipeptides (excluding anserine and carnosine). Box plots demonstrating the organ-specific distribution of the dipeptides Ala-Ala, Ala-Gln, Asp-Gln, Glu-Ser, Gly-Asp, His-Ser, showing median as bold line with standard deviation and ● indicating outliers (*n* = 6). The two glutamine dipeptides (Ala-Gln and Asp-Gln) and Ala-Ala were the major dipeptides in liver, Glu-Ser in muscle and spleen, Gly-Asp in spleen and thymus and His-Ser in muscle.

Ala-Ala, Ala-Gln, Asp-Gln, Glu-Ser, Gly-Asp and His-Ser were also detected at high concentrations in the liver, spleen and thymus (Figure 4). In the liver, two glutamine-containing dipeptides Ala-Gln (11,551 ± 2968 fmol/mg tissue) and Asp-Gln (2402 ± 673 fmol/mg tissue) and the alanine-containing dipeptide Ala-Ala (1777 ± 1013 fmol/mg tissue) were present at high concentrations. In spleen and thymus, the two dipeptides Glu-Ser (14,137 ± 1393 and 1238 ± 227 fmol/mg tissue) and Gly-Asp (5803 ± 442 and 6013 ± 667 fmol/mg tissue, respectively) were highly abundant. Dipeptide concentrations were higher in brown compared to white adipose tissue with particular high concentrations of Gly-Asp in brown tissue (1192 ± 117 fmol/mg). In the muscle, two serine-containing dipeptides Glu-Ser (2159 ± 1280 fmol/mg tissue) and His-Ser (2100 ± 670 fmol/mg tissue) were highly abundant dipeptides next to anserine and carnosine.

**Figure 5:**
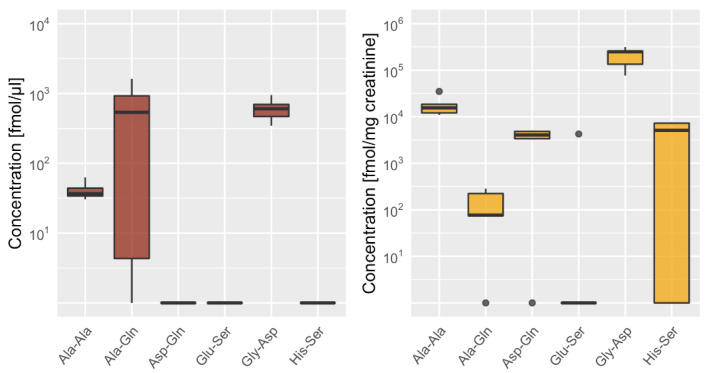
Distribution of the six most abundant dipeptides (except carnosine and anserine) in urine and serum. Box plot showing the distribution of Ala-Gln, Gly-Asp, Glu-Ser, His-Ser, Ala-Ala and Asp-Gln in urine in fmol/mg creatinine (*n* = 5) and serum in fmol/µL (*n* = 6), showing median as bold line with standard deviation and ● indicating outliers.

In serum, dipeptide concentrations per ml were several magnitudes lower as compared to mg tissue; anserine was most abundant, followed by γ-Glu-ε-Lys, Ala-Gln and Gly-Asp. In urine, anserine was most abundant, followed by Gly-Asp, Gly-Pro, Pro-Gly and γ-Glu-ε-Lys (Figure 5; see Supplementary Table S2).

### 2.4. Tissue-Specific Distribution of Isomeric Dipeptides Forms

**Figure 6:**
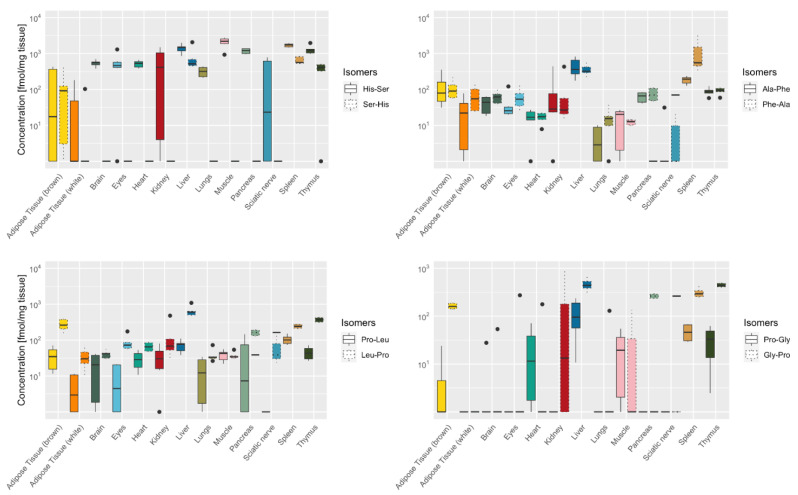
Distribution of four stereoisomeric dipeptides in murine tissue. Box plot showing the organ-specific concentration of four structural isomers (Ala-Phe/Phe-Ala; His-Ser/Ser-His; Pro-Leu/Leu-Pro; Gly-Pro/Pro-Gly), showing median as a bold line with standard deviation and ● indicating outliers (*n* = 6).

We then analyzed whether the distribution pattern of isomeric forms of dipeptides differs between organs. Four dipeptides were present at different isoforms, i.e., with specific amino acids either at the C- or N-position of the dipeptide quantified, Ala-Phe/Phe-Ala, His-Ser/Ser-His, Pro-Leu/Leu-Pro, and Gly-Pro/Pro-Gly (Figure 6).

Both isoforms composed of alanine and phenylalanine (Ala-Phe/Phe-Ala) showed a similar distribution pattern across the organs with exception of the spleen with 6-fold higher Phe-Ala concentrations compared to Ala-Phe (1209 ± 1213 and 185 ± 43 fmol/mg tissue). In all organs, dipeptides with proline in the C-terminus (Gly-Pro and Leu-Pro) were present at higher concentrations than dipeptides with N-terminal proline (Pro-Gly and Pro-Leu). His-Ser was the abundant isomer in pancreas, sciatic nerve, muscle, kidney, heart, and brain, while the concentrations of the isomeric dipeptide Ser-His were low.

In urine, three of the four isomeric dipeptide counterparts were present, Ala-Phe/Phe-Ala at similar concentrations, and Leu-Pro and Gly-Pro at higher concentrations then Pro-Leu and Pro-Gly, respectively (Figure 5). In serum, none of the isomeric dipeptides were detected in both isoforms.

### 2.5. Tissue-Specific Amino Acid Profiles Relative to Dipeptide Profiles

**Figure 7:**
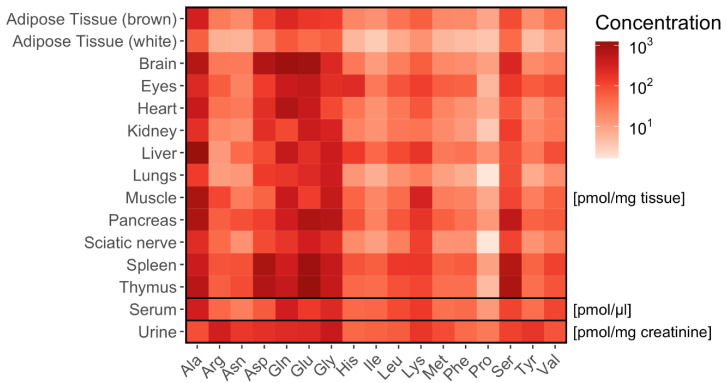
Distribution of amino acids in murine tissues and biofluids. Heat map showing the concentrations for 17 amino acids concentration (*n* = 6) in murine tissues (adipose tissue, brain, eyes, heart, kidney, liver, lungs, muscle, nervus ischiadicus, pancreas, spleen and thymus) and biofluids (serum, urine). Mean values are given in pmol/mg tissue (*n* = 6). In serum, the concentrations are given in pmol/µL and in urine in pmol/mg creatinine (*n* = 5).

A total of 17 Amino acids were quantified in murine tissue (Figure 7; Supplementary Table S6).

**Figure 8:**
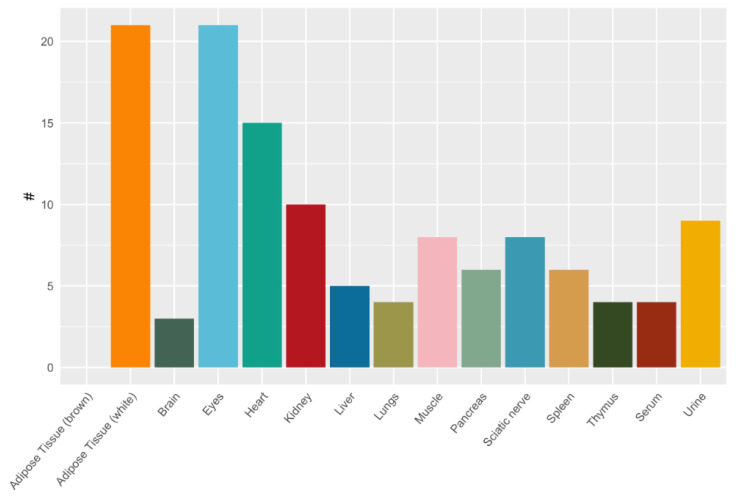
Correlation of dipeptides and respective amino acids in murine tissues and biofluids.

We then related tissue-specific amino acid profiles with respective dipeptide profiles. The correlation (correlation factor *p* > 0.7) between dipeptides and their respective amino acids was highly tissue-specific and depending on the properties of the dipeptides, determined by the localization of the amino acid in either C- or N-position of the dipeptide (Figure 8).

Displayed is the number of dipeptides correlating with their respective amino acid positioned in different organs and biofluids. In white adipose tissue 21 dipeptides correlated (*p* > 0.7) with either one (4 dipeptides) or both (17 dipeptides) corresponding amino acids (Supplementary Table S7). In contrast, in brown adipose tissue no such correlations were found. In eye samples 21 and in the heart 15 dipeptides correlated with one or both corresponding amino acids. In other tissues 3 to 10 dipeptides correlated with at least one of the corresponding amino acids (Supplementary Table S7; Figure 8).

Several tissue dipeptide concentrations correlated with the respective amino acid concentrations only when the amino acid was present at the C-terminus of the dipeptide. This applied to the dipeptides containing Asp at C-terminal position (Gly-Asp), His (Ala-His, Gly-His, Ser-His), Pro (Ala-Pro, Leu-Pro) and Ser (Glu-Ser, His-Ser). Dipeptides containing the same amino acids at N-terminal position were not correlated to the respective amino acid tissue concentrations (Asp-Gln, His-Ser, Pro-Gly, Pro-Leu, Ser-Ala).

Tissue concentrations of alanine were highly correlated with alanine-containing dipeptides independent of the isomeric dipeptide form. In most tissues alanine concentrations correlated with dipeptides containing alanine in the N-position (such as Ala-Ala, Ala-Gln, Ala, Gly, Ala-Phe, Ala-Pro, Ala-Tyr) and in the C-position (such as Ser-Ala). The dipeptides containing Glu (γ-Glu-ε-Lys, Glu-Glu, Glu-Ser, Ala-Glu, Gly-Glu), Gly (Gly-Asp, Gly-Glu, Gly-His, Gly-Phe, Gly-Pro, Als-Gly, Pro-Gly), Phe (Phe-Ala, Ala-Phe, Gly-Phe, Tyr-Phe) and Tyr (Tyr-Ala, Ala-Tyr, Val-Tyr) in either N- or C-position correlated with their respective amino acids in many tissues.

In serum, alanine concentrations correlated with Ala-containing dipeptides independent of the alanine position in the dipeptide (Ala-Ala, Ala-Glu, Ala-Gly, Ala-Pro). In urine, only few correlations were found (Supplementary Table S7). Dividing the dipeptides according to their amino acid depending on charge (positive, negative, neutral), their side-chain polarity (acidic/basic polar/non-polar), and structure components (aromatic, aliphatic, combined) did not yield specific organ distribution patterns.

## 3. Discussion

Amino acids have a central role in metabolism and their intracellular changes contribute to the pathogenesis of various diseases, such as phenylketonuria. The role of amino acid transporters and their role in interorgan distribution of amino acids has been well described [8]. The tissue-specific distribution of carnosine and anserine with different carnosine-to-anserine ratios in the organs has been described in detail [13,14], but little is known about the distribution of other dipeptides and their relationship to amino acid metabolism. Moreover, it remains unknown to what extent organs can maintain dipeptides to release amino acids in adaption to the metabolic situation.

Using a newly developed AccQ-Tag™ derivatization-based UPLC-MS/MS method, we found that dipeptides are widely distributed in the murine body with a tissue-specific distribution pattern. A total of 30 of the 36 dipeptides investigated could be detected in the analyzed organs and body fluids with high sensitivity (with LODs of <100 amol on column) and reliability (with respect to recovery and variability). Some dipeptides, such as carnosine, anserine, Ala-Ala, Asp-Glu and Glu-Ser were present in all organs while others, such as Arg-Phe, were below the detection limit in all organs. The highest concentration of anserine and carnosine was found in the muscle, and the highest concentration of Gln-containing dipeptides in the liver, spleen and thymus. The dipeptide pattern of structural dipeptide isomers (His-Ser and Ser-His; Ala-Phe and Phe-Ala; Pro-Leu und Leu-Pro; Pro-Gly and Gly-Pro) indicates that the position of the amino acid in N- or C-terminus is important for their organ-specific distribution.

The high dipeptide concentration in the liver is consistent with its high metabolic activity and central role in protein and amino acid metabolism [15]. In particular, Gln-containing dipeptides are present in the liver, indicating their role as glutamine donor. Gln concentration is mainly controlled by the liver and skeletal muscle and is the most abundant and versatile amino acid in the body. Gln is of fundamental importance to intermediary metabolism and interorgan nitrogen exchange via ammonia transport between tissues and pH homeostasis [8,16]. Under disease conditions the liver consumes more Gln. To increase endogenous Gln concentrations, stable glutamine releasing dipeptides such as Ala-Gln and Gly-Gln are used [17,18,19,20] instead of free Gln. Parenteral supplementation with Gln dipeptides significantly reduced hospital mortality and infectious complication rates in critically ill patients [18,19,21] and short-term supplementation of peritoneal dialysis fluids resulted in restored stress responses and improved immune competence of peritoneal cells [20]. Besides the liver, skeletal muscle also generates a major portion of the total pool of free amino acids and is a major store for glutamine. In the skeletal muscle, not Gln-containing dipeptides but carnosine, anserine and Ser-containing and, to some extent, Gly-containing dipeptides were present in high concentrations. Ser can easily be converted to Gly, a precursor for creatine, heme, purines, and glutathione [22] and has a central role in muscle function. Ser availability and metabolism is dysregulated in aged skeletal muscles [23]. In spleen and thymus, amino acid availability, synthesis, and catabolism are highly interrelated aspects of immune responses and may reflect to the high dipeptide concentrations in those tissues. In particular, the importance of Gln [16] and Arg [24] in immune response have been described. Dipeptide and amino acid abundance were much higher in brown compared to white adipose tissue which seems to reflect the higher metabolic activity of brown compared to white adipose tissue [25].

The analysis of isomers showed that the distribution of the dipeptides is related, at least for some dipeptides, to their structure. In all organs, dipeptides with proline in the C-terminus (Gly-Pro and Leu-Pro) were present at higher concentrations than dipeptides with N-terminal proline (Pro-Gly and Pro-Leu). In studies looking at the intestinal uptake of dipeptides and their distribution in the blood after feeding, it has been demonstrated that the dipeptide structure is a major factor for dipeptide appearance in the peripheral blood [5,6]. Determination of resistance of dipeptides against hydrolysis in human colon carcinoma Caco-2 cells, showed that dipeptides such as anserine, carnosine, γ-glutamyl-dipeptides, and Gly-Asp are highly resistant against hydrolysis [5] which seems to contribute to the high concentration of those dipeptides in urine and serum in our mice. From these findings and our dipeptide panel it can be assumed that the isomeric structure contributes to the distribution of dipeptides within the different organs.

It is noticeable that the structure of the dipeptides seems to have also an influence on amino acid availability. Although amino acids do not only originate from dipeptides but also show a high metabolic flux, some trends can be described. While some dipeptides (containing Ala, Glu, Gly, Phe and Tyr) are correlated with their respective amino acids independent on the position in N- or C-terminus, other dipeptides (containing Asp, His, Pro or Ser) correlate with their respective amino acids more often when positioned in C-terminus. Among the latter, there were more polar amino acids (such as Asp, His and Ser), but whether this is an important factor needs further investigation. Effects of other amino acid properties such as side-chain polarity or class were not found. Some amino acids, such as Gln, hardly correlated with their corresponding dipeptides, which might be explained by low stability of Gln and its high metabolic turnover.

Even though it has not yet been systematically studied, there is increasing evidence that dipeptides can have functions beyond their roles as amino acid donors [26,27]. Several studies could demonstrate that carnosine and anserine have specific dipeptide functions such as antioxidant activity [14,28] which cannot be explained by their corresponding amino acids alone. For example, treatment with carnosine or anserine prevented alterations of renal function in diabetic (db/db) mice [29,30,31]. Additionally, for proline-containing dipeptides the biological activities seem to be not only based on the free amino acid alone [1]; for Tyr-containing dipeptides [32] an inhibitory effect on ACE activity has been demonstrated, and Tyr-Asp improves plant tolerance to oxidative stress by directly interfering with glucose metabolism [33]. Gly-containing dipeptides can provide osmoregulation in preimplantation embryos [34]. In kidney of streptotozin-induced diabetic mice and in diabetic patients, γ-Glu-ε-Lys seems to contribute to alterations in epithelial cell viability and tubular atrophy [35]. Alterations in dipeptide concentrations have been described for cancer [36], Parkinson disease [1], Alzheimer [37], Huntington disease [38], bronchopulmonary disease [39] or diabetes [35,40] and further studies in respective disease states are of interest.

Our optimized AccQ-Tag™ UPLC-MS/MS method allows simultaneous, fast and sensitive detection of a large panel of diverse dipeptides with high recovery rates. The sample processing is very rapid and simple compared to other methods using PITC, which requires a drying and reconstitution step [5,6]. In addition to the many advantages of this method, there are also some limitations. Some dipeptides were not detectable in the selected sample types and dipeptides containing multiple amine groups generally showed lower intensities and therefore higher detection limits compared to dipeptides with only one amino group. Of note is that not all theoretically possible dipeptide combinations and their isomeric isoforms were identified by the panel, thus our novel findings cannot be generalized.

## 4. Materials and Methods

### 4.1. Chemicals

Dipeptide standard substances were purchased from Sigma-Aldrich (Schnelldorf, Germany). AccQ-Tag™ Ultra Derivatization Kit was obtained from Waters (Eschborn, Germany). Mobile phases and wash solutions were prepared with UHPLC-MS grade acetonitrile (Merck, Darmstadt, Germany), water and formic acid (Biosolve, Valkenswaard, Netherlands). Dipeptide standard substances were dissolved in water to a concentration of 5 mM, except for Gly-Phe, Tyr-Phe and His-Leu which were acidified using ultra-pure HCl. The stock solutions of all dipeptides were diluted to a final concentration of 100 µM. Further dilutions were prepared in water and norleucine as internal standard was added to each dilution step with 10 µM final concentration.

### 4.2. Organ Harvest

Non-fasting C57BL/6N mice were euthanized at the age of 15 weeks by inhalation of CO2 with following exsanguination via vena cava. Organs were perfused with 0.9% NaCl via the left ventricle route. Vena cava and abdominal aorta were dissected at the lumbar vertebra region to guarantee an appropriate flow of perfusion solution. Organs, blood and urine were cryopreserved in liquid nitrogen immediately after extraction.

### 4.3. Sample Treatment

Amino acids and dipeptides were extracted from pulverized frozen mouse tissue using 0.1 mL ice-cold 0.1 M HCl (with 10 µM norleucine as internal standard) per 20 mg weight. Liquid samples were diluted 30-fold with 0.1 M HCl (with 10 µM norleucine as internal standard). The samples were then labeled with AccQ-Tag™ according to the manufacturer’s protocol with slight modifications as follows: 35 µL borate buffer were mixed with 5 µL sample or calibration standard, 20 µL AccQ-Tag reagent were added. After incubation at room temperature for one minute the samples were heated for 10 min at 55 °C. Afterwards 440 µL ddH_2_O was added and the samples were centrifuged at 16,400× *g* for 15 min at room temperature. The supernatant was transferred into TruView vials (Waters) and used for analysis.

### 4.4. Chromatographic Conditions

Separation was performed using an ACQUITY UPLC I-class PLUS system (Waters) and an ACQUITY HSS T3 column (100 mm × 2.1 mm, 1.8 µm, Waters) heated to 40 °C. Solvent A was 0.1% formic acid in water, solvent B 0.1% formic acid in acetonitrile (UHPLC-MS quality). Chromatographic parameters are depicted in Table 2, the injection volume was 5 µL. Samples were kept at 10 °C during measurements.

### 4.5. Mass Spectrometry Conditions

Detection was carried out with a QTRAP 6500+ mass spectrometry system (Sciex, Darmstadt, Germany) equipped with an ESI IonDrive™ Turbo V Source using scheduled MRM mode for the detection of different dipeptides, parameters are listed in Table 3. Compound-specific details on MRM transitions, potentials and retention times are listed in Table 1. Analyst^®^ 1.7 (Sciex) was used as system operation software. Evaluation and analysis of data was performed using SciexOS^®^ software (Sciex, Version 1.4.0.18067) and AutoPeak algorithm. Quantification parameters can be found in Supplementary Table S1.

### 4.6. Method Validation

Linearity was analyzed using serial dilutions of calibration standards between 50 µM and 5 nM. At least 5-point calibration curves of standard mixtures were used for quantification. A maximum coefficient of variation (CV) of 20% in calibration standards was allowed. Determination of recovery rates was carried out by 30-fold diluted calibration standard with 0.1 M HCl (with 10 µM norleucine) and spiking of extracted samples of lung tissue with serial dilutions of calibration standards. Intra-assay precision was assessed by measuring the 50 fmol calibration standards in triplicates at least, and inter-assay precision was determined by derivatizing and analyzing the 50 fmol calibration standard on three different days out of eight measurements. The limit of detection (LOD) was defined as three times the signal-to-noise ratio (S/N), the limit of quantification (LOQ) was set as ten times the S/N-ratio according to analytical standard guidelines. The full list of dipeptides that could be analyzed according to these criteria is shown in Table 1.

### 4.7. Amino Acid and Creatinine Quantification

Amino acids were determined as previously described by Weger et al. [41]. The panel of 17 amino acids included alanine (Ala), arginine (Arg), asparagine (Asn), aspartate (Asp), glutamine (Gln), glutamate (Glu), glycine (Gly), histidine (His), Isoleucine (Ile), leucine (Leu), lysine (Lys), methionine (Met), phenylalanine (Phe), proline (Pro), serine (Ser), tyrosine (Tyr) and valine (Val). Creatinine determination in murine urine samples was performed with the creatinine assay kit (Abcam, ab204537) following precisely the manufacturer’s protocol.

### 4.8. Statistical Analysis

Data were obtained from at least five biological replicates. Heatmaps (Figure 2 and Figure 7) show the mean. Violin plot (Figure 3) and box plots (Figure 4, Figure 5 and Figure 6) show the median as a bold line with standard deviation (SD). Statistical analysis was performed with R version 4.0.4 (15 February 2021) with the following packages: readxl_1.3.1, stringr_1.4.0, dplyr_1.0.5, tibble_3.1.1, ggplot2_3.3.3, ggpubr_0.4.0 and extrafont_0.17.

## 5. Conclusions

To control organ function and to react to metabolic needs in the cells, coordinated interactions of proteins and amino acids between organs are necessary [42]. With our UPLC-MS/MS method, we demonstrated a tissue-specific distribution of dipeptides. Distribution of dipeptides and their relation to the respective amino acids concentrations is influenced by the position of the amino acid in either N- or C-terminus of the dipeptide. The now established method is reliable for simultaneous quantification of various dipeptides which is smoothly expandable to more compounds of interest and forms a versatile platform for enhanced studies of dipeptides. As dipeptides appear to have a function beyond that of a pure amino acid source, the administration of dipeptides may offer new therapeutic options.

## Figures and Tables

**Table 1:** Mass transitions (Q1 and Q3), retention time (RT), declustering potential (DP), collision energy (CE) and collision cell exit potential (CXP) for each analyte with peak numbers.

Peak No.	Analyte	Q1	Q3	RT	DP	CE	CXP
1	Seryl-Histidine (Ser-His)	413.0	171.1	2.08	61	39	20
2	Glycyl-Histidine (Gly-His)	383.0	213.1	2.15	31	21	12
			171.1			35	20
3	Histidyl-Serine (His-Ser)	413.0	171.1	2.31	46	31	16
4	Carnosine (β-Ala-His)	397.0	171.1	2.62	56	43	18
			110			51	14
5	Serinyl-Glutamine (Ser-Gln)	403.9	171.1	2.65	126	53	10
6	Alanyl-Histidine (Ala-His)	397.0	171.1	2.71	46	35	20
7	Anserine (β-Ala-3methyl-His)	411.0	171.1	2.73	131	39	10
8	Glycyl-Aspartic Acid (Gly-Asp)	361.0	171.1	2.84	31	31	16
9	Aspartyl-Glutamine (Asp-Gln)	431.9	171.1	2.97	46	27	18
10	Glutamyl-Serine (Glu-Ser)	404.9	171.1	3.16	131	35	20
11	Glycyl-Glutamic Acid (Gly-Glu)	374.9	171.1	3.18	146	25	10
12	Histidylalanine (His-Ala)	397.0	171.1	3.23	51	31	14
13	Alanyl-Glutamine (Ala-Gln)	387.9	171.1	3.41	106	27	14
14	Alanyl-Glycine (Ala-Gly)	317.0	171.1	3.53	31	33	12
15	Seryl-Alanine (Ser-Ala)	347.0	171.1	3.6	51	33	18
16	Glutamyl-Glutamic Acid (Glu-Glu)	447.0	171.1	3.61	41	41	18
17	Prolyl-Glycine (Pro-Gly)	343.0	171.1	3.73	66	35	20
			89			103	10
18	Glycyl-Sarcosine (Gly-Sar)	317.0	171.1	3.77	56	31	20
			116			77	12
19	Alanyl-Glutamic Acid (Ala-Glu)	388.9	171.1	3.79	126	23	10
20	Alanyl-Alanine (Ala-Ala)	331.0	171.1	4.05	36	31	10
21	Glycyl-Proline (Gly-Pro)	343.0	171.1	4.71	86	31	20
22	γ-Glutamyl-ε-Lysine (γ-Glu-ε-Lys)	616.0	171.1	5.41	31	49	12
23	Alanyl-Tyrosine (Ala-Tyr)	422.9	171.1	5.46	21	37	20
24	Alanyl-Proline (Ala-Pro)	357.0	171.1	5.61	36	33	20
25	Leucyl-Histidine (Leu-His)	439.0	156	5.64	26	33	18
			171.1			33	18
26	Tyrosyl-Alanine (Tyr-Ala)	422.9	171.1	5.65	21	39	20
27	Histidyl-Leucine (His-Leu)	439.1	110	5.97	26	43	12
			171.1			31	10
28	Arginyl-Phenylalanine (Arg-Phe)	492.1	171.1	6	36	47	20
29	Glycyl-Phenylalanine (Gly-Phe)	392.9	171.1	6.98	16	37	20
30	Valyl-Tyrosine (Val-Tyr)	451.0	171.1	7	26	39	16
31	Prolyl-Leucine (Pro-Leu)	399.1	171	7.22	46	39	20
			229.2			23	14
32	Alanyl-Phenylalanine (Ala-Phe)	407.0	171.1	7.48	56	37	20
			242.1			21	16
33	Phenylalanyl-Alanine (Phe-Ala)	407.0	171.1	7.66	61	37	10
			120.1			29	14
34	Internal Std—Norleucine (2-Aminohexanoic acid)	302.0	171.1	8.21	81	25	16
35	Aspartame (Asp-Phe-methylester)	465.1	171.1	8.77	21	37	20
36	Tyrosyl-Phenylalanine (Tyr-Phe)	499.0	171.1	8.86	26	41	20
37	Leucyl-Proline (Leu-Pro)	399.1	171.1	8.97	56	37	20
			229.2			21	20

**Table 2:** Chromatographic gradient.

Time (min)	Flow (mL/min)	%A	%B
Initial	0.5	98	2
0.50	0.5	98	2
11.50	0.5	70	30
11.51	0.5	10	90
13.00	0.5	10	90
13.10	0.5	98	2
16.00	0.5	98	2

**Table 3:** Mass spectrometry parameters.

Parameter	Details
Scan type	MRM
Polarity	positive
MRM detection window (s)	60
Target scan time per sMRM (s)	0.6
Curtain Gas (CUR)	40
Collision Gas	Medium
IonSpray Voltage (IS)	5500
Temperature (TEM)	550
Ion Source Gas 1 (GS1)	70
Ion Source Gas 2 (GS2)	70
Entrance Potential (EP)	10

## Data Availability

All data associated with this study are provided in the main figures and tables and in the supplementary information.

## Supplementary Materials

The following are available online at https://www.mdpi.com/article/10.3390/ijms22189979/s1, Table S1: Quantification parameters in SciexOS, Table S2: Calibration range of dipeptides (fmol on column) and precision for 50 fmol; Table S3: Recovery in spiked lung tissue samples (mean ± SD; *n* = 3); Table S4: Recovery of analytes in standard mixtures (mean ± SD; *n* = 3); Table S5: Dipeptide concentrations in tissue, serum and urine (mean ± SD; *n* = 6); Table S6: Amino acid concentrations in tissue, serum and urine (mean ± SD; *n* = 3); Table S7: Correlation (>0.7) of dipeptides with their corresponding amino acids in either C- or N-position.
