# Supplementary material for: A Novel UPLC-MS/MS Method Identifies Organ-Specific Dipeptide Profiles
Source: Int J Mol Sci. 2021 Sep 15;22(18):9979. doi: 10.3390/ijms22189979 (PMC8465603; doi:10.3390/ijms22189979)
Supplement: Supplementary file 1 [file ijms-22-09979-s001.zip › ijms-1372756-supplementary.pdf]

International Journal of
Molecular Sciences

MDPI

Supplementary Material

Supplementary table 1. Quantification parameters in SciexOS

Parameter	Details
Integration Algorithm	AutoPeak
Peak selection	By expected retention time
Preprocessing	Low smoothing
	Noise filter
Minimum peak height	100
Minimum signal/noise	3
Regression parameter	Area
Regression type	Linear through zero
Weighting type	None
Internal standard	Norleucine

Supplementary table 2. Calibration range of dipeptides (fmol on column) and precision for 50 fmol

Analyte	Lower Limit	Upper Limit	slope	r^2	%CV intra-assay (n=3)	%CV inter-assay (n=8)
**Ala-Ala**	0.1	500	0.68665	0.99	1.50	3.4
**Ala-Gln**	1.25	500	0.07204	0.99	4.40	4.9
**Ala-Glu**	0.1	500	0.25489	0.99	3.00	8
**Ala-Gly**	0.1	500	0.5183	0.99	3.20	4.7
**Ala-His**	1.25	1250	0.01365	0.99	9.10	14.5
**Ala-Phe**	0.1	500	0.86063	0.99	0.40	1.8
**Ala-Pro**	0.25	500	0.77599	0.99	0.80	6.1
**Ala-Tyr**	0.1	500	0.46913	0.99	2.00	8.9
**Anserine**	2.5	1250	0.00407	0.99	9.60	12
**Arg-Phe**	2.5	1250	0.01338	0.98	18.50	27.2
**Asp-Gln**	0.5	500	0.02695	0.99	2.60	6.1
**Aspartame**	0.1	500	0.94842	0.99	1.10	1.1
**Carnosine**	2.5	1250	0.01212	0.99	11.70	13
**Glu-Glu**	0.1	500	0.26133	0.99	2.20	7.7
**Glu-Ser**	0.1	500	0.27531	0.99	3.60	8.1
**Gly-Asp**	0.1	500	0.38197	0.99	0.60	3.2
**Gly-Glu**	0.1	500	0.19695	0.99	3.10	5.1
**Gly-His**	1.25	500	0.02151	0.99	1.70	20.6
**Gly-Phe**	0.1	500	0.83350	0.99	1.00	9.3
**Gly-Pro**	1.25	2500	0.47691	0.99	1.10	1
**Gly-Sar**	0.1	2500	0.46406	0.99	2.00	4.9
**His-Ala**	2.5	1250	0.00646	0.99	10.20	9.4
**His-Leu**	1.25	1250	0.01749	0.99	3.40	6.6
**His-Ser**	2.5	1250	0.00395	0.99	12.40	15.6
**Leu-His**	2.5	1250	0.01121	0.99	0.70	13.2
**Leu-Pro**	0.1	500	1.23841	0.99	1.60	3.8
**Phe-Ala**	0.1	500	0.71722	0.99	1.90	3
**Pro-Gly**	0.1	500	0.34212	0.99	2.40	6.1
**Pro-Leu**	0.1	500	0.78262	0.99	3.20	3.8
**Ser-Ala**	0.25	500	0.59395	0.99	1.50	3.8
**Ser-Gln**	0.25	500	0.06133	0.99	6.20	5.6
**Ser-His**	1.25	1250	0.00617	0.99	8.70	23.2
**Tyr-Ala**	0.1	500	0.52569	0.99	0.60	10.7
**Tyr-Phe**	0.1	500	0.52747	0.99	0.60	12.2
**Val-Tyr**	0.1	500	0.61847	0.99	2.10	6.5
**γ-Glu-ε-Lys**	1.25	250	0.0076	0.99	25.30	25.6

Supplementary table 3. Recovery in spiked lung tissue samples (mean±SD; n=3)

Analyte	Level 1			Level 2			Level 3		
**Ala-Ala**	83.40	±	0.61	90.54	±	0.49	90.31	±	1.12
**Ala-Gln**	79.50	±	6.82	87.79	±	0.97	89.50	±	1.25
**Ala-Glu**	79.72	±	3.67	89.73	±	1.96	90.05	±	0.59
**Ala-Gly**	74.67	±	2.96	86.31	±	1.43	87.54	±	1.33
**Ala-His**	64.02	±	6.03	74.71	±	3.65	81.21	±	2.26
**Ala-Phe**	81.63	±	0.80	90.17	±	1.05	90.91	±	0.59
**Ala-Pro**	78.73	±	0.72	88.27	±	0.44	91.69	±	1.93
**Ala-Tyr**	80.39	±	1.39	88.86	±	0.34	88.97	±	0.75
**Anserine**				104.49	±	3.52	85.06	±	2.36
**Arg-Phe**	83.10	±	2.67	81.22	±	3.00	87.84	±	0.36
**Asp-Gln**	71.82	±	4.86	86.10	±	4.01	88.89	±	0.71
**Aspartame**	85.42	±	0.63	93.73	±	0.45	93.83	±	0.82
**Carnosine**	71.58	±	25.24	78.83	±	8.28	78.80	±	1.07
**γ-Glu-ε-Lys**	92.80	±	14.22	82.05	±	3.89	80.28	±	2.00
**Glu-Glu**	81.86	±	3.93	90.09	±	0.57	91.70	±	1.09
**Glu-Ser**	87.27	±	3.24	90.80	±	2.21	89.32	±	0.44
**Gly-Asp**	102.57	±	2.81	99.71	±	1.02	100.57	±	2.59
**Gly-Glu**	79.58	±	3.46	86.90	±	1.16	90.22	±	0.08
**Gly-His**	75.68	±	2.12	82.03	±	1.31	85.14	±	1.58
**Gly-Phe**	83.74	±	1.70	91.56	±	1.66	93.18	±	0.53
**Gly-Pro**	72.45	±	0.09	87.00	±	0.13	92.19	±	0.43
**Gly-Sar**	79.03	±	2.02	90.51	±	0.67	93.25	±	0.54
**His-Ala**	66.12	±	15.40	77.98	±	4.13	80.44	±	2.13
**His-Leu**	75.99	±	5.57	85.74	±	1.77	85.13	±	1.65

**His-Ser**	95.28	±	14.25	78.85	±	4.20	88.25	±	1.42
**Leu-His**	80.27	±	1.38	83.00	±	5.08	80.16	±	1.39
**Leu-Pro**	83.91	±	1.14	91.59	±	0.44	92.93	±	0.29
**Phe-Ala**	83.16	±	1.88	89.37	±	0.86	91.09	±	0.17
**Pro-Gly**	92.84	±	3.44	94.61	±	1.34	96.90	±	0.40
**Pro-Leu**	84.07	±	0.47	90.98	±	0.60	90.77	±	0.20
**Ser-Ala**	84.52	±	2.06	89.59	±	1.06	91.01	±	0.87
**Ser-Gln**	76.20	±	7.32	86.05	±	1.01	87.72	±	1.06
**Ser-His**	84.69	±	6.09	82.70	±	0.69	85.05	±	1.28
**Tyr-Ala**	80.91	±	2.65	90.71	±	1.13	91.19	±	1.37
**Tyr-Phe**	79.00	±	0.98	89.84	±	1.23	91.23	±	0.68
**Val-Tyr**	81.38	±	2.39	90.16	±	1.30	90.48	±	0.78

Level 1: 2.5 fmol on column
Level 2: 25 fmol on column
Level 3: 250 fmol on column

Supplementary table 4. Recovery of analytes in standard mixtures (mean±SD; n=3)

Analyte	Level 1			Level 2			Level 3		
**Ala-Ala**	101.0	±	0.7	105.5	±	0.1	101.7	±	0.1
**Ala-Gln**	98.1	±	1.9	103.5	±	0.6	102.1	±	0.5
**Ala-Glu**	103.5	±	0.7	107.2	±	0.9	104.5	±	0.7
**Ala-Gly**	100.0	±	1.8	101.4	±	0.7	102.8	±	1.0
**Ala-His**	81.3	±	2.1	89.8	±	0.8	90.7	±	1.5
**Ala-Phe**	98.8	±	0.3	103.6	±	0.4	100.1	±	0.4
**Ala-Pro**	94.7	±	0.4	93.6	±	0.6	100.1	±	0.6
**Ala-Tyr**	94.5	±	0.1	100.1	±	0.3	97.5	±	0.0
**Anserine**	74.9	±	5.5	89.2	±	2.8	88.1	±	4.6
**Arg-Phe**	100.4	±	1.3	101.3	±	1.9	99.3	±	0.5
**Asp-Gln**	101.3	±	2.1	104.0	±	1.3	103.5	±	2.8
**Aspartame**	99.6	±	0.2	103.1	±	0.4	101.9	±	0.1
**Carnosine**	85.7	±	3.7	85.6	±	4.6	87.9	±	1.6
**γ-Glu-ε-Lys**	87.3	±	3.5	92.8	±	3.0	90.0	±	1.9
**Glu-Glu**	103.6	±	1.4	106.6	±	0.2	105.7	±	0.3
**Glu-Ser**	105.1	±	1.3	109.7	±	0.7	107.1	±	1.0
**Gly-Asp**	99.2	±	0.7	103.5	±	0.8	99.7	±	1.2
**Gly-Glu**	96.2	±	0.5	102.4	±	1.2	96.6	±	0.2
**Gly-His**	86.9	±	0.8	91.8	±	1.8	91.4	±	0.8
**Gly-Phe**	97.4	±	0.7	102.3	±	0.7	99.1	±	0.2
**Gly-Pro**	93.9	±	0.2	98.3	±	0.7	95.1	±	0.1
**Gly-Sar**	94.5	±	0.5	97.7	±	0.1	94.9	±	0.5
**His-Ala**	85.6	±	1.4	91.0	±	1.3	94.1	±	3.5
**His-Leu**	90.2	±	1.2	93.9	±	0.7	94.2	±	1.0
**His-Ser**	92.6	±	0.4	94.4	±	3.7	97.8	±	0.9
**Leu-His**	92.5	±	2.2	92.0	±	3.9	95.2	±	1.7
**Leu-Pro**	98.2	±	0.3	102.4	±	0.6	99.0	±	0.5
**Phe-Ala**	98.8	±	0.5	103.9	±	1.2	100.7	±	0.7
**Pro-Gly**	119.8	±	1.2	116.9	±	0.5	118.9	±	0.5
**Pro-Leu**	109.3	±	1.5	110.8	±	0.5	110.4	±	0.7
**Ser-Ala**	105.0	±	1.2	108.5	±	0.5	105.0	±	0.9
**Ser-Gln**	101.0	±	1.4	105.7	±	2.1	104.0	±	0.4
**Ser-His**	94.1	±	0.4	99.0	±	1.2	99.3	±	0.7
**Tyr-Ala**	99.6	±	1.4	103.6	±	0.5	99.7	±	0.8
**Tyr-Phe**	96.9	±	0.3	102.9	±	0.3	99.7	±	0.2
**Val-Tyr**	97.6	±	0.3	103.0	±	0.5	98.9	±	0.5

Level 1: 33.3 fmol on column
Level 2: 83.3 fmol on column
Level 3: 166.7 fmol on column

Supplementary table 5. Dipeptide concentrations in tissue, serum and urine (mean±SD; n=6)

	Brown Adipose tissue	White Adipose tissue	Brain	Eyes	Heart	Kidney	Liver
Dipeptide				fmol/mg tissue			
Ala-Ala	477±368	93±71	161±62	131±93	107±35	351±455	1777±1013
Ala-Gln	332±146	54±47	84±31	258±348	171±162	205±310	11551±2968
Ala-Glu	523±369	160±122	278±106	140±92	72±22	191±258	760±319
Ala-Gly	305±127	69±49	76±100	103±71	57±18	243±279	855±346
Ala-His	872±318	b.d.	b.d.	b.d.	24±58	56±138	498±297
Ala-Phe	130±124	28±30	42±21	41±40	17±10	104±170	458±270
Ala-Pro	237±75	32±22	52±19	45±30	66±16	143±203	815±225
Ala-Tyr	866±433	49±44	189±105	141±91	41±18	170±224	391±77
Anserine	46564±33746	9777±4735	33853±10340	74156±48011	115762±40282	17615±8858	35249±9029
Asp-Gln	282±426	b.d.	295±243	b.d.	b.d.	273±445	2402±673
Carnosine	9434±13552	988±560	97644±125007	15827±10218	7136±5048	4280±2739	268±
γ-Glu-ε-Lys	561±445	291±131	307±241	452±332	310±163	395±397	1167±687
Glu-Glu	235±127	58±47	353±53	146±123	121±35	228±246	795±533
Glu-Ser	802±454	157±118	575±158	604±555	86±27	271±362	831±179
Gly-Asp	1192±117	311±137	1976±245	1130±717	1499±394	1664±748	3309±783
Gly-Glu	220±66	51±29	271±36	211±176	338±69	190±134	899±222
Gly-His	697±423	b.d.	260±226	71±552	268±378	144±211	2125±1211
Gly-Phe	297±117	25±16	58±18	54±33	18±7	56±68	309±124
Gly-Pro	165±28	b.d.	9±22	46±112	30±73	212±354	465±138
His-Ser	187±209	60±92	546±109	540±427	521±113	608±637	1420±395
Leu-Pro	282±108	36±23	42±14	86±46	68±22	136±172	640±236
Phe-Ala	114±77	68±49	64±29	66±45	17±6	98±165	367±148
Pro-Gly	5±10	b.d.	5±11	b.d.	25±30	b.d.	120±91
Pro-Leu	38±25	5±6	21±20	10±11	30±17	35±29	71±26
Ser-Ala	467±270	83±61	150±39	133±72	114±32	349±518	640±533
Ser-Gln	252±100	71±46	92±21	196±84	26±21	195±254	822±325
Ser-His	122±154	17±42	b.d.	b.d.	b.d.	b.d.	782±645
Tyr-Ala	50±38	7±9	12±3	13±14	8±3	31±50	91±40
Tyr-Phe	142±150	21±18	54±27	34±21	7±4	84±117	222±137
Val-Tyr	80±85	19±13	43±13	36±36	14±4	57±60	133±48

	Lungs	Muscle	Pancreas	Sciatic nerve	Spleen	Thymus	Serum	Urine (n=5)
**Dipeptide**		**fmol/mg tissue**			**fmol/µl**		**fmol/mg creatinine**	
**Ala-Ala**	31±4	209±88	146±42	108±40	861±131	414±123	41±12	18442±9769
**Ala-Gln**	15±15	77±52	196±94	478±502	937±290	612±316	627±634	132±118
**Ala-Glu**	32±8	86±6	166±36	54±24	1539±392	1138±286	4±6	862±382
**Ala-Gly**	36±6	57±17	119±44	197±155	4293±1018	346±60	155±342	2492±1491
**Ala-His**	b.d.	b.d.	b.d.	b.d.	409±136	375±66	b.d.	b.d.
**Ala-Phe**	5±5	15±12	64±20	5±13	185±43	89±22	b.d.	44±61
**Ala-Pro**	29±18	35±6	392±152	56±26	537±86	559±149	14±25	3352±1259
**Ala-Tyr**	7±4	274±65	95±38	46±37	365±53	153±19	b.d.	85±117
**Anserine**	24690±5920	5399365±2120970	14683±3497	61090±21894	202932±13481	100476±10319	6659±1278	1416808±343416
**Asp-Gln**	b.d.	b.d.	209±340	b.d.	1295±293	2270±595	b.d.	3476±2058
**Carnosine**	938±380	2303395±1438991	1216±1172	29093±11654	5862±1292	3861±974	256±398	16284±16426
**γ-Glu-ε-Lys**	552±328	348±287	341±298	343±294	1455±504	969±788	861±567	36471±51450
**Glu-Glu**	54±10	361±112	264±70	109±32	733±148	807±139	3±8	4706±2194
**Glu-Ser**	101±36	2159±1280	345±165	307±253	14137±1393	1238±227	b.d.	853±1908
**Gly-Asp**	1104±260	2211±461	3248±306	666±209	5803±442	6013±667	611±215	207245±98258
**Gly-Glu**	130±24	605±151	962±169	85±24	707±89	1214±227	64±37	14205±7113
**Gly-His**	b.d.	232±177	330±193	b.d.	2375±910	612±384	b.d.	b.d.
**Gly-Phe**	9±2	42±8	119±32	18±7	483±87	125±15	29±9	143±95
**Gly-Pro**	22±53	41±63	266±33	b.d.	309±70	442±65	b.d.	134138±53721
**His-Ser**	323±104	2100±670	1188±202	329±369	1715±203	1293±367	b.d.	4013±3792
**Leu-Pro**	38±17	37±9	165±40	71±67	243±38	377±87	17±12	7459±3510
**Phe-Ala**	16±12	12±3	77±35	7±11	1209±1213	93±20	b.d.	87±50
**Pro-Gly**	b.d.	23±22	b.d.	b.d.	48±20	33±23	b.d.	41287±20329
**Pro-Leu**	16±16	39±13	47±60	b.d.	105±31	46±19	b.d.	1354±1913
**Ser-Ala**	56±16	47±8	214±49	49±23	1189±167	422±63	b.d.	141±133
**Ser-Gln**	16±17	26±29	114±33	16±39	913±157	377±72	b.d.	1618±582
**Ser-His**	b.d.	b.d.	b.d.	b.d.	669±212	375±209	b.d.	b.d.

**Tyr-Ala**	4±3	10±2	8±9	17±22	105±21	111±20	b.d.	b.d.
**Tyr-Phe**	8±4	4±3	138±34	18±11	115±73	80±34	b.d.	b.d.
**Val-Tyr**	7±4	25±8	53±13	14±12	149±44	44±14	3±7	3203±1165

b.d. = below detection limit

Supplementary table 6. Amino acid concentrations in tissue, serum and urine (mean±SD; n=3)

	**Brown Adipose tissue**	**White Adipose tissue**	**Brain**	**Eyes**	**Heart**	**Kidney**	**Liver**
**Amino acid**	**pmol/mg tissue**						
**Ala**	336±57	68±43	694±196	269±163	468±105	212±81	1253±279
**Arg**	29±5	7±3	31±3	69±37	37±10	22±9	14±6
**Asn**	19±3	7±3	30±7	25±12	31±10	18±9	45±14
**Asp**	105±16	24±13	706±58	144±55	216±73	224±96	100±22
**Gln**	254±31	81±44	1103±134	464±274	732±229	120±61	670±390
**Glu**	209±106	50±24	1493±793	519±223	472±138	477±268	205±67
**Gly**	140±17	63±25	273±127	223±136	104±29	350±193	422±123
**His**	21±3	6±3	31±6	276±225	35±11	26±12	145±40
**Ile**	16±3	4±1	12±2	33±17	15±3	17±9	50±13
**Leu**	35±5	9±4	27±4	86±47	33±8	36±19	101±23
**Lys**	61±10	17±8	62±15	135±64	77±24	40±20	171±50
**Met**	20±3	6±3	20±4	67±32	23±8	21±11	31±8
**Phe**	18±2	5±3	17±3	59±35	15±5	14±7	37±11
**Pro**	20±19	8±9	22±19	17±21	16±15	6±7	55±58
**Ser**	96±12	49±24	277±96	156±79	74±21	159±95	91±28
**Tyr**	18±3	5±2	19±4	75±48	15±6	23±12	32±11
**Val**	38±4	10±4	27±5	96±40	32±6	35±17	92±23

	**Lungs**	**Muscle**	**Pancreas**	**Sciatic nerve**	**Spleen**	**Thymus**	**Serum**	**Urine (n=5)**
**Amino acid**	**pmol/mg tissue**						**pmol/µl**	**pmol/mg creatinine**
**Ala**	139±24	883±400	787±131	220±39	413±71	611±85	361±110	119±96
**Arg**	11±2	110±23	66±29	44±11	81±14	62±8	47±6	443±351

**Asn**	13±3	28±7	89±16	16±6	86±12	98±11	27±7	165±68
**Asp**	142±16	55±16	126±39	103±28	834±173	717±59	91±59	209±100
**Gln**	164±35	448±121	377±62	169±34	374±49	476±72	346±79	252±130
**Glu**	237±35	136±35	819±213	365±118	1111±224	1233±110	174±96	278±152
**Gly**	387±52	510±202	688±100	201±30	468±58	485±50	246±60	462±132
**His**	14±2	53±9	81±11	18±5	80±11	47±4	47±10	50±18
**Ile**	7±1	21±4	24±5	11±1	61±11	42±5	50±6	65±35
**Leu**	16±3	42±9	75±6	25±4	151±26	85±9	99±11	70±29
**Lys**	25±7	327±107	172±24	123±13	155±29	111±21	149±35	195±129
**Met**	10±2	29±10	61±15	16±5	55±10	40±4	38±4	104±44
**Phe**	7±2	24±7	35±5	15±3	67±11	40±5	44±4	49±27
**Pro**	3±8	35±40	78±86	3±8	43±62	28±60	32±28	59±37
**Ser**	91±12	114±31	521±53	114±24	660±107	779±50	120±47	127±49
**Tyr**	8±2	26±8	65±37	15±4	54±11	39±7	39±7	174±49
**Val**	17±2	56±13	68±6	29±5	121±19	80±7	110±9	84±21

b.d. = below detection limit

Supplementary table 7. Correlation (>0.7) of dipeptides with their corresponding amino acids in either C- or N-position

Dipeptide	Organ	AA in N-terminus	Correlation	AA in C-terminus	Correlation
**Gly-Asp**	Adipose Tissue (white)	Gly	0,90	Asp	0,92
**γ-Glu-ε-Lys**	Adipose Tissue (white)	Glu	0,70	Lys	0,79
**Glu-Ser**	Adipose Tissue (white)	Glu	0,82	Ser	0,88
**Ala-Ala**	Adipose Tissue (white)	Ala	0,97	Ala	0,97
**Gly-Glu**	Adipose Tissue (white)	Gly	0,94	Glu	0,93
**Ala-Gln**	Adipose Tissue (white)	Ala	0,87	Gln	0,65
**His-Ser**	Adipose Tissue (white)	His	0,96	Ser	0,77
**Ala-Gly**	Adipose Tissue (white)	Ala	0,96	Gly	0,95
**Leu-Pro**	Adipose Tissue (white)	Leu	0,94	Pro	0,71
**Glu-Glu**	Adipose Tissue (white)	Glu	0,98	Glu	0,98
**Ala-Glu**	Adipose Tissue (white)	Ala	0,93	Glu	0,95
**Ala-Pro**	Adipose Tissue (white)	Ala	0,92	Pro	0,58
**Ser-Ala**	Adipose Tissue (white)	Ser	0,81	Ala	0,91

**Ser-Gln**	Adipose Tissue (white)	Ser	0,73	Gln	0,65
**Val-Tyr**	Adipose Tissue (white)	Val	0,86	Tyr	0,90
**Ala-Tyr**	Adipose Tissue (white)	Ala	0,92	Tyr	0,71
**Phe-Ala**	Adipose Tissue (white)	Phe	0,81	Ala	0,81
**Gly-Phe**	Adipose Tissue (white)	Gly	0,92	Phe	0,93
**Ala-Phe**	Adipose Tissue (white)	Ala	0,94	Phe	0,90
**Tyr-Phe**	Adipose Tissue (white)	Tyr	0,66	Phe	0,80
**Tyr-Ala**	Adipose Tissue (white)	Tyr	0,89	Ala	0,92
**Pro-Gly**	Brain	Pro	0,12	Gly	0,84
**Gly-Glu**	Brain	Gly	0,92	Glu	0,54
**Gly-Phe**	Brain	Gly	0,79	Phe	0,31
**Gly-Asp**	Eyes	Gly	0,99	Asp	0,88
**Gly-Pro**	Eyes	Gly	0,99	Pro	0,00
**γ-Glu-ε-Lys**	Eyes	Glu	0,82	Lys	0,79
**Glu-Ser**	Eyes	Glu	0,97	Ser	0,97
**Ala-Ala**	Eyes	Ala	0,98	Ala	0,98
**Gly-Glu**	Eyes	Gly	0,99	Glu	0,98
**His-Ser**	Eyes	His	0,89	Ser	0,87
**Ala-Gly**	Eyes	Ala	0,76	Gly	0,73
**Leu-Pro**	Eyes	Leu	0,95	Pro	0,28
**Glu-Glu**	Eyes	Glu	0,98	Glu	0,98
**Ala-Glu**	Eyes	Ala	0,97	Glu	0,96
**Ala-Pro**	Eyes	Ala	0,97	Pro	0,21
**Ser-Ala**	Eyes	Ser	0,96	Ala	0,98
**Ser-Gln**	Eyes	Ser	0,92	Gln	0,95
**Val-Tyr**	Eyes	Val	0,99	Tyr	0,97
**Ala-Tyr**	Eyes	Ala	0,95	Tyr	0,88
**Phe-Ala**	Eyes	Phe	0,87	Ala	0,90
**Gly-Phe**	Eyes	Gly	0,96	Phe	0,95
**Ala-Phe**	Eyes	Ala	0,98	Phe	0,98
**Tyr-Phe**	Eyes	Tyr	0,92	Phe	0,95
**Tyr-Ala**	Eyes	Tyr	0,97	Ala	0,95
**Gly-Asp**	Heart	Gly	0,91	Asp	0,95
**Glu-Ser**	Heart	Glu	0,94	Ser	0,90
**Ala-Ala**	Heart	Ala	0,78	Ala	0,78
**Gly-Glu**	Heart	Gly	0,86	Glu	0,83
**Ala-Gly**	Heart	Ala	0,80	Gly	0,57
**Leu-Pro**	Heart	Leu	0,86	Pro	0,58
**Glu-Glu**	Heart	Glu	0,85	Glu	0,85
**Ala-Glu**	Heart	Ala	0,94	Glu	0,86

**Ser-Ala**	Heart	Ser	0,97	Ala	0,82
**Val-Tyr**	Heart	Val	0,64	Tyr	0,70
**Ala-Tyr**	Heart	Ala	0,67	Tyr	0,80
**Phe-Ala**	Heart	Phe	0,73	Ala	0,99
**Gly-Phe**	Heart	Gly	0,86	Phe	0,79
**Ala-Phe**	Heart	Ala	0,91	Phe	0,87
**Tyr-Ala**	Heart	Tyr	0,84	Ala	0,28
**Gly-Asp**	Kidney	Gly	0,98	Asp	0,76
**Gly-Glu**	Kidney	Gly	0,78	Glu	0,78
**His-Ser**	Kidney	His	0,80	Ser	0,98
**Leu-Pro**	Kidney	Leu	0,72	Pro	-0,31
**Glu-Glu**	Kidney	Glu	0,79	Glu	0,79
**Val-Tyr**	Kidney	Val	0,71	Tyr	0,39
**Phe-Ala**	Kidney	Phe	0,75	Ala	0,36
**Gly-Phe**	Kidney	Gly	0,64	Phe	0,78
**Ala-Phe**	Kidney	Ala	0,32	Phe	0,71
**Tyr-Phe**	Kidney	Tyr	0,31	Phe	0,76
**Gly-Asp**	Liver	Gly	0,89	Asp	0,88
**γ-Glu-ε-Lys**	Liver	Glu	0,81	Lys	0,46
**His-Ser**	Liver	His	0,55	Ser	0,86
**Leu-Pro**	Liver	Leu	0,79	Pro	0,50
**Glu-Glu**	Liver	Glu	-0,71	Glu	-0,71
**Ala-Pro**	Liver	Ala	0,86	Pro	0,71
**Ala-Phe**	Liver	Ala	-0,84	Phe	-0,19
**Gly-Asp**	Lungs	Gly	0,81	Asp	0,34
**Gly-Glu**	Lungs	Gly	0,76	Glu	0,85
**Glu-Glu**	Lungs	Glu	0,74	Glu	0,74
**Val-Tyr**	Lungs	Val	0,70	Tyr	0,85
**Gly-Pro**	Muscle	Gly	0,90	Pro	0,08
**Pro-Gly**	Muscle	Pro	0,34	Gly	0,75
**Ala-Ala**	Muscle	Ala	0,93	Ala	0,93
**Gly-Glu**	Muscle	Gly	0,82	Glu	0,30
**Ala-Gly**	Muscle	Ala	0,64	Gly	0,73
**Gly-His**	Muscle	Gly	0,85	His	0,84
**Ala-Pro**	Muscle	Ala	0,79	Pro	0,26
**Val-Tyr**	Muscle	Val	0,73	Tyr	0,66
**Ala-Phe**	Muscle	Ala	-0,70	Phe	-0,81
**γ-Glu-ε-Lys**	Pancreas	Glu	-0,42	Lys	-0,81
**Gly-His**	Pancreas	Gly	0,76	His	0,62
**Ala-Pro**	Pancreas	Ala	0,83	Pro	0,37

**Ser-Ala**	Pancreas	Ser	-0,73	Ala	0,11
**Val-Tyr**	Pancreas	Val	0,24	Tyr	0,81
**Ala-Tyr**	Pancreas	Ala	0,91	Tyr	0,65
**Tyr-Ala**	Pancreas	Tyr	0,55	Ala	0,83
**γ-Glu-ε-Lys**	Sciatic nerve	Glu	0,87	Lys	0,49
**Ala-Ala**	Sciatic nerve	Ala	0,91	Ala	0,91
**Gly-Glu**	Sciatic nerve	Gly	-0,09	Glu	0,85
**Ala-Gln**	Sciatic nerve	Ala	0,77	Gln	0,62
**Glu-Glu**	Sciatic nerve	Glu	0,73	Glu	0,73
**Ala-Glu**	Sciatic nerve	Ala	0,87	Glu	0,56
**Ser-Ala**	Sciatic nerve	Ser	0,28	Ala	0,77
**Val-Tyr**	Sciatic nerve	Val	0,75	Tyr	0,41
**Gly-Asp**	Serum	Gly	-0,80	Asp	-0,66
**Ala-Ala**	Serum	Ala	0,86	Ala	0,86
**Ala-Gly**	Serum	Ala	0,92	Gly	0,72
**Ala-Glu**	Serum	Ala	0,82	Glu	0,61
**Ala-Pro**	Serum	Ala	0,86	Pro	0,38
**Gly-Pro**	Spleen	Gly	0,95	Pro	0,58
**Glu-Ser**	Spleen	Glu	0,61	Ser	0,74
**Ala-Pro**	Spleen	Ala	0,82	Pro	0,84
**Ala-His**	Spleen	Ala	-0,05	His	0,87
**Gly-Phe**	Spleen	Gly	0,52	Phe	0,86
**Ser-His**	Spleen	Ser	-0,26	His	0,71
**Gly-Pro**	Thymus	Gly	0,87	Pro	0,36
**Phe-Ala**	Thymus	Phe	0,78	Ala	0,23
**Gly-Phe**	Thymus	Gly	0,84	Phe	0,77
**Ser-His**	Thymus	Ser	0,12	His	0,78
**Gly-Asp**	Urine	Gly	0,60	Asp	0,88
**γ-Glu-ε-Lys**	Urine	Glu	-0,21	Lys	0,76
**Pro-Gly**	Urine	Pro	0,74	Gly	0,31
**Gly-Glu**	Urine	Gly	0,56	Glu	0,77
**Ala-Gln**	Urine	Ala	0,47	Gln	0,98
**His-Ser**	Urine	His	0,81	Ser	0,79
**Ala-Pro**	Urine	Ala	0,00	Pro	0,87
**Val-Tyr**	Urine	Val	-0,84	Tyr	0,59
**Ala-Phe**	Urine	Ala	0,76	Phe	0,30
